# Wine Aging Technology: Fundamental Role of Wood Barrels

**DOI:** 10.3390/foods9091160

**Published:** 2020-08-23

**Authors:** Maria Carpena, Antia G. Pereira, Miguel A. Prieto, Jesus Simal-Gandara

**Affiliations:** 1 Nutrition and Bromatology Group, Analytical and Food Chemistry Department, Faculty of Food Science and Technology, University of Vigo, Ourense Campus, E-32004 Ourense, Spain; carpenarodriguezm@gmail.com (M.C.); antia.gonzalez.pereira@uvigo.es (A.G.P.); 2 Centro de Investigação de Montanha (CIMO), Instituto Politécnico de Bragança, Campus de Santa Apolonia, 5300-253 Bragança, Portugal

**Keywords:** wine aging, oxidative phase, barrels, oak species, phenolic and aromatic compounds

## Abstract

The aging of wines is a process used to preserve wine but also to enhance its properties. It is a process of great interest, mainly because of the additional properties it adds to wines and because of its economic implications. Historically, barrels have been employed for centuries for preserving and aging wine due to their resistance and relative impermeability. In general terms, the wine aging process can be divided into two phases: oxidative and reductive aging. Oxidative aging traditionally takes place in barrels while reductive phase occurs in the bottle. During both processes, oxygen plays a fundamental role as well as other factors, for instance: temperature, light, bottle position, microbial growth or storage time. Likewise, during the aging process, a series of chemical reactions take place influencing the composition and organoleptic profile of wine. At this point, oxidative aging in barrels is a fundamental step. Barrels are directly involved in the produced changes on wine’s composition due to the transference of oxygen and phenolic and aromatic compounds from wood to wine. This way, barrels act as an active vessel capable of releasing compounds that affect and improve wine’s characteristics. Regarding, the importance of barrels during aging process, some attention must be given to the species most used in cooperage. These species are conventionally oak species, either French or American. However, other non-conventional species are currently being studied as possible wood sources for the production of wines, such as chestnut robinia or other oak species. In the last decades, new approaches have been developed for barrel aging to find new alternatives more suitable, affordable and feasible to sanitize the process, such as other materials different from wood or the use of wood chips, which is regulated since 2006 by the EU. However, even though some of them have shown promising data, barrels are currently the most used technology for the oxidative stage of table wines aging.

## 1. Introduction

### 1.1. History

The conservation and aging of wine is a historical practice carried out throughout millennia by many civilizations. According to archaeological findings, the first large-scale production of wine, began in Mesopotamia at least at ≈5400 before common era (BCE) and the earliest wine transport vessels were developed by Greeks and Romans around 2000 BCE. Fermentation and maturation were done on earthenware jars and amphorae, which were cheap and very common, but had a series of inconveniences, as they were fragile, heavy and difficult to handle. With the advancement of technology and in order to solve these problems, barrels were developed [1,2]. Wooden barrels begun being widely used as containers for oil and wine more than 2000 years ago in Northern Europe, under control of the Roman Empire since clay was not as easily available in those regions [3,4]. Since its invention, barrels have stored, transported and aged not only wine but also another variety of liquids such as beer, whiskeys and other liquors [3]. Oak and chestnut wood were the most frequently used for cooperage in the 16th century due to their resistance, flexibility and relative impermeability [5]. Today, they continue to be the only species allowed by the OIV [6]. Oak is still the major used wood, followed by chestnut whereas other woods such false acacia or cherry species are still being studied. These last ones have been more widely used since the last decades [7]. However, wines can be aged in a traditional way using other materials different from wood such as cement or steel, but these are not so appreciated as they do not confer to the beverages the unique aromas that wood barrels transfer to the products [8]. The development of these alternative materials is mainly due to an increase in global demand for wood barrels, especially those of oak, with the aim of reproducing the chemical and physical processes experienced by the wines during their aging in barrels [2]. The use of wood barrels for wine aging involves long times (from 6 to 18 months in barrels) and high economic costs, so various techniques have been investigated to obtain similar products. In the last decades several alternative aging techniques have been developed, including the addition of pieces of wood of various shapes and sizes; a method that has risen in popularity in Europe since it was approved and regulated by the European Commission and OIV [6,9].

### 1.2. Special Interest of Aging Wines

Throughout the aging process, a series of reactions take place resulting in changes in chemical composition and organoleptic properties. These changes will give rise to modifications in its final quality, due to complex aroma from wood, increasing stability and clarification [2]. Two variables are fundamental in the aging process: the aging time and the quality of the barrel wood. Aging process also varies depending on the type of wine. Regarding red wines, the aging is formed by two distinct stages: wood phase (oxidative) and bottle phase (reductive) [10]. White wines may also be aged, but for many years, it was believed that this process was not adequate because white table wine suffered a decrease on their quality as wood masked the white wine components and contributed to oxidative processes [11]. However, the use of wood for white wine production and aging is also a tendency in modern wine market to produce full body or fortified wines with a more complex aroma, due to the results obtained in recent studies where brown pigments do not represent a problem [12]. This process has been applied through a bâtonnage technique, also known as less stirring, as a way to extract flavor, aroma and texture in white wines. In the wood phase, the type of wood of the barrel is extremely important, since inside it, wine is oxidized. The barrel allows the passage of very low amounts of oxygen, a process called oxygenation. At the same time, several chemical reactions take place inside it, alongside a transfer of different phenolic compounds (PC) from the wood to the wine and vice versa [13].

The most relevant reactions that take place during aging are anthocyanin and tannin condensation, oxidation and the release of molecules from wood to wine (PC, aromatic compounds (AC)). All these compounds combined with the aromatic terpenoids and polyphenols already present in wine are responsible for the overall complex flavor and aromas [14,15]. These reactions contribute to wine clarification, modifying its astringency and developing its tertiary aroma as a result of the presence and interaction of all the AC present at the aging time end [16]. However, this process also presents difficulties such as adequacy of time, development of undesirable microorganisms or further undesirable oxygenation, which lead to varying levels of volatile acidity or excessive astringency [15]. Depending on the time that wine remains in the barrel (3 to 12 months), three types of wines can be distinguished, from least to most valuable and aged: aging, reserve and large reserve [17]. Even so, the “reserve” naming is subjected to national regulations, and in some countries, it may not be correlated with barrel aging time.

Therefore, the aging of wine is a process widely used in oenology to provide an added value to the wine. As mentioned before, this process has been traditionally carried out in barrels made of oak, but the growing demand and the increasing interest on the process itself has led to the implementation of other methods (use of wood chips, wine lees or research on other wood species for cooperage) and also to the investigation of the chemical and physical processes that wines undergo during their stay in the barrels. In general terms, it has led to an increase of the research at this area [18].

### 1.3. Economic Data

In general, five main economic agents are involved in the production of wines and thus also in aged ones: non-associated viticulturists who do not produce, non-associated vintners that produce or grow, associated winemakers, wholesalers and aging supervisors. This variety of agents makes possible a better production and to target specific sectors of the wide spectrum of consumers [19]. All these economic agents allowed worldwide wine exports to reach 10 × 10^3^ million liters in 2019, showing a 0.3% decrease compared to previous years. Despite being a smaller volume, earnings increased by 0.9% [20]. As for the importance of each country, the evolution of the world market in recent years has made Italy (152 × million L) and Spain (86 million L), surpassing France (19.3 million L), as the world′s leading wine exporters. Despite this, France accounts for the most profitable winemaking industry (443 million €), at a price that almost reaches 7 €/L, despite its negative production trend [21]. Consumers associate the aging of the wines in barrels with quality, which has a truly scientific basis given the highly appreciated notable changes in flavor [22]. Furthermore, factors such as fashion, year of harvest or terroir influence consumers’ preferences making them more prone to buying aged wines and expending more on them [23]. The market for this type of products has been progressively increasing around the world. Proof of this is the increase in the number of wineries that produce aging wines in La Rioja (Spain), which went from 63 in 1983 to 386 in 2012. In addition, it can also be seen that the market for wines from that region is mostly aged wines, observing that in 2012, of every 100 bottles sold, approximately 45 were of young wine and 55 aged wines (37 aging, 16 reserves and 2 large reserves) [19].

Regarding oak trade economic data, as of 2018 the United Nations Commodity Trade Statistics Database reports that France accounted more than 180 million $ total as revenue for oak lumbers exports, while the US is the export leader, having reached more than 1,160 million $ [24]. The main oak species used in cooperage are *Quercus petraea* and *Q. robur* from European countries, namely from French forests, and *Q. alba* from U.S.A. forests, usually referred as French and American oak, respectively, as species are conventionally named for the geographical area where they are mostly produced. This report just provides data on oak lumber trade where aside from cooperage, oak wood is highly valued for furniture manufacture, for example. Nevertheless, these data show that these two producers are leading oak production and trade. Therefore, it reveals the worth of economic activity that this field represents, and the great demand for this wood, and it explains the surge of new and alternative methodologies and techniques to obtain aged wines [25].

### 1.4. Wine Requirements for the Aging Process

The composition of wine is complex and changes continuously during aging. Wine maturation is mainly influenced by the oxidation process, which includes change of color, loss of varietal character and the development of aldehydic aroma. The oxidation depends on different factors such as pH, temperature, concentration of dissolved oxygen and phenolic composition [26]. It has been proved that higher pH and temperature improves the process. However, the type and concentration of PC and their susceptibility to oxidation are determinant on this matter [27]. Not all wines can be subjected to an aging process since several aptitudes are required. The chemical parameters that must be met are set out in different laws according to the origin of the wine, especially in wines subjected to appellation of origin. These chemical parameters are mostly related to the phenolic maturity of grapes, which determinate the concentration of PC that, as mentioned, are determinant during the aging process [28]. Taking Spain as an example, according to a study carried out by the Andalusian government, in order to age a red wine in an optimal manner, wines must have at least a total polyphenol index (TPI) above 60 mg GAE/g (as it prevents oxidation and protects color), high content in selected polyphenols, more than 800 mg/L of anthocyanins and more than 3 g/L tannins and not very low pH (>3.50) [29]. The alcoholic strength should be around 12%, a minimum total acidity of 4.5 g/L expressed in tartaric acid and a volatile acidity of less than 13.3 mEq/L (it can be exceeded if the alcoholic strength is higher) [29]. In the same way, the Castilla La Mancha government demands that red wines with appellation of origin “Valdepeñas” have similar requirements, but demands a lower maximum acidity (4 g/L) and even sets the maturation conditions needed to produce this type of wines. Generally, these parameters can be applied to wines submitted to a Mediterranean climate. [30]. However, several studies have researched different times of harvesting in order to assess the changing chemical profile of grapes and its consequences during the aging process [31,32]. Similar values have been found in terms of acidity (range 3–5) [33,34] pH (>3.50) [34] or anthocyanins content (>800 mg/L) [31], among others, in wines of different origins. In any case, these values are just a reference as some factor such as soil or climate fluctuates from one geographical area to another and so, values do as well. For instance, grapes with thick skins, produced in dry areas and with little irrigation will have less water and thus a higher proportion of sugar, acids and phenols. Therefore, by decreasing the amount of water, the potential for aging is increased [35]. In general, the parameters usually studied to define harvesting time are the content of sugars, total acidity (in terms of tartaric acid), pH, alcoholic strength and phenolic content, especially in anthocyanins and tannins.

On the other hand, in terms of preventing microbial growth of undesirable or spoilage microorganisms, the most widely employed technique for preventing microbial spoilage as well as an excessive oxidation of wine is by the addition of sulfur dioxide. In this regard, maximum legal SO_2_ levels vary from one country to another. For non-sparkling or liquor wines, the European Union sets a maximum 150 mg/L in red wines and 200 mg/L in white wines (with some exemptions depending on the total level of residual sugars), whereas the USA increases the general limit to 350 mg/L [36,37]. This is a fundamental step before aging as microbial growth of these microorganisms can alter the organoleptic attributes of wine during the aging process [37]. Furthermore, to carry out the aging process correctly, it is recommended to perform a cleaning phase before putting the wine in the barrel to avoid possible secondary aging defects.

### 1.5. Importance of Oxygen Transfer

While oxidation usually involves negative implications, the term oxygenation alludes to the liberation of oxygen and its contact with wine, a technique used in oenology to improve wine properties (color, aroma, texture). Oxygen modulates yeast fermentation, improves wine stability and increases its complexity by modifications on its aromatic profile. It also plays an important role in the determination of some reactions as tannins and anthocyanins polymerization, SO_2_ reduction or deletion or ethanol to acetaldehyde oxidation [38,39]. These effects will vary in intensity depending on the oxygenation conditions, temperature, microbial strains used and the composition of the wine. All these parameters are essential to effectively manage wine oxygen exposure [15,40]. Moreover, it is expected that more rationalized oxygen management strategies will allow a decrease in the use of SO_2_, a prescription that has been demanded by the major food and health organizations worldwide [41]. Particularly, SO_2_ naturally present or afterwards added acts as antioxidant and antimicrobial but produces allergic reactions in some individuals and so its use is limited. Its reaction with oxygen is usually slow and plays a marginal role in SO_2_ antioxidant activity. Nevertheless, the ability of SO_2_ to reduce H_2_O_2_ to H_2_O and reconvert quinones into phenols, transform it into an effective wine antioxidant. Therefore, if less oxygen is added to the wine (by means of rationalized strategies), less SO_2_ would be necessary to control the oxidative processes that occur during winemaking [41,42]. Therefore, oxygen plays a fundamental role in the vinification process and occurs in various stages, particularly during the fermentation and aging of wines [43]. Polyphenols present in wine are the major consumers of oxygen, and they are known essentially for their antioxidant capacity. Thus, given their increased presence in red wines, in general, they will require a higher oxygenation time in comparison with aged white wines. However, the time will also depend on the final sensory wine profile requested and others factors such as wine variety [44]. Once the wine has been transferred to the bottle, further oxygenation is generally inconvenient since it could lead to undesired degradation of sulfur volatile and PC. It could also accelerate the evolution and maturation of red wine through oxidative and acetaldehyde-mediated reactions [45,46].

Several studies have investigated the changes produced by the oxygenation process. In a study conducted with Nebbiolo of wines treated with different doses of oxygen (7, 14, 21 and 28 mg/L total intake), it was observed that storage promotes a decrease in color intensity and anthocyanins, while there was an increase in polymeric pigments and minor aldehydes. Nevertheless, color differences between oxygen doses were modest whereas oxygen played a fundamental role in free acetaldehyde content as higher doses increase its concentration [40]. Different studies were carried out in this regard. One of them studied Pinotage wine under different ripening time conditions and carrying out in all of them monthly oxygenations at two doses of oxygen (2.5 and 5.0 mg O_2_/L/month) for zero, two, four and six months. It was observed that the best results were obtained at low doses after two months according to the sensory characteristics, while high doses and exposure time showed harmful sensory effects. All these treatments had the disadvantage of decreasing the total antioxidant capacity, which was avoided through the elaboration of a new protocol that consisted of doses of 1.0 mg of O_2_/L in discrete doses every two weeks for two months [47]. These studies exemplify the amount of oxygen needed in the aging wine process. In the case of wood barrels’ oxygenation, the dose of received oxygen depends on the oxygen transfer rate (OTR) of the barrel. In a recent study the role of oxygen during the aging process on barrels and bottles showed that those wines submitted to an aging process in barrels with higher OTR had more ability for consuming oxygen while taking less time, resulting in an increase in color intensity [39]. The parameters that will determine the necessary quantity of oxygen when monitoring is available will be presence of sulfur compounds (namely SO_2_), the initial wine composition (that comes determined by concentration of anthocyanins and tannins and plant characteristics such as varietals, vineyards and vintages) and final destiny (which will be influenced in turn by timing and temperature) [38]. In the case of wood barrels, the OTR will depend on the chosen species, its geographical origin or cooperage process [39]. These characteristics will result in a different porosity of woods (coarseness of grain) and also in the allowance of a greater or lesser transfer of oxygen.

## 2. Barrels: Active Vessels. Manufacture, Function and Effects

**Figure 1:**
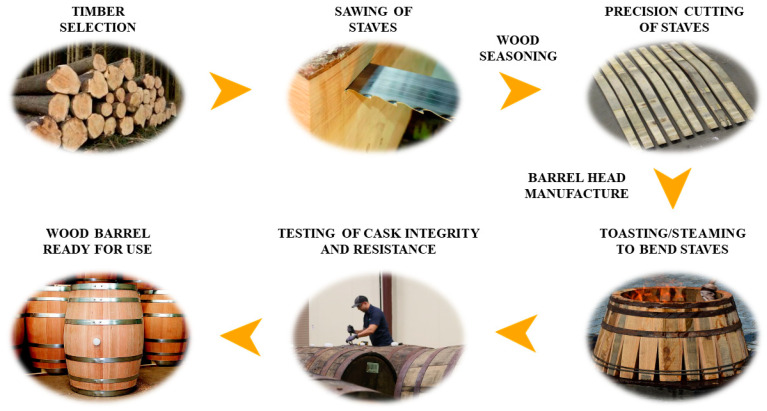
Barrel manufacturing process.

Regarding all the previous information, this article is aimed at providing a revision of the actual knowledge on the oxidative aging phase of wine, particularly, regarding barrel aging. Therefore, the main aspects about barrels will be developed in the following sections as well as the compounds present in wood and the several species used in cooperage. Barrels have functions greater than those of serving as a container. The use of barrels allows to separate the sediments from the wine, provides tannins, allows oxygenation and stabilizes color. During aging, wood releases compounds to the wine and contributes to the improvement of its organoleptic properties. All this contributes to the aging of wine by adding complexity, flavor and longevity to the resulting beverage. Therefore, wine barrel acts as an active vessel that releases chemical compounds into the wine, improving its physical, chemical and sensory properties. Depending on its origin, age, thickness, uses, roasting and the time that wine is left inside, the acquired properties are different. As compounds that can be extracted from wood are finite, factors such as the composition of the wine, maintenance applied to the barrels and fermentation affect the shelf life of barrels, decreasing the extraction rate and the amounts of compounds extracted as the barrel is used in successive batches. Moreover, undesirable or spoilage microorganisms may grow and develop biofilms in the inner side of the barrel and especially in the junction gaps between staves, which can be extremely resilient to sanitation procedures [48]. The main chemical changes due to contact with the wood are the exchange of PC, especially ellagitannins (ET), between the wood and the stored wine and AC [49,50,51]. Oak wood is the most extensively used for wine-aging barrels’ manufacture. Oak is usually chosen due to its hardness, permeability, contribution of characteristic aromas, certain ability to inhibit molds and yeasts growth, its mechanical properties and usage tradition. Another important factor is the cost, being those of French oak more expensive than the American ones due to losses involved because of its more irregular grain and greater porosity. This characteristic entails that wood has to be divided instead of sawn which leads to a lower yield from the wood [52]. Other factors of great importance happen during the manufacturing process (Figure 1) including size, since large barrels account for a lower PC exchange to the wine due to the lower ratio of wood surface/volume of wine. This seems to be less relevant in the case of old barrels, as their PC exchange is already lowered [49]. Another important factor is barrel toasting. Toasting changes both the quantity and the quality of the extractable substances in the oak wood of barrels, being possible to differentiate barrel toasting levels by analyzing the volatile and semi volatile compounds resulting from the thermal degradation of oak [53]. This process provides smoked flavor as the toasting intensity increases but causes the thermodegradation of several compounds: carbohydrates, resulting in furanic compounds; lignin or hemicellulose with the consequent volatile phenols formations and acids, which by dehydration result in oak lactones [54]. For example, phenyl ketones are the result of the lignans breakdown, so their concentration continuously increases during toasting [53]. In addition, toasting can be carried out on different conditions (high, medium, low) and be influenced by factors such as the origin of the wood compounds or the watering process during toasting. For instance, a recent study showed that, in general, wine watering process during toasting results in a lower concentration of ET [54]. Even so, the composition is quite changeable during the toasting process. On this regard, a study conducted a real time mass spectrometry monitoring of the process, highlighting the variable nature of oak wood chemistry as individual oak boards of the same origin, moisture content and density showed different values under similar conditions [55]. Other authors studied the influence of the oak species and found that the volatile composition evolved similarly between different species during toasting (French, American and Spanish), but important quantitative differences were found in American species with respect to European species [56]. Besides the added flavor, this heat treatment also eases the ending of the wood staves so that they can be molded to a barrel shape [49].

The main and essential characteristic that should be noted regarding barrels is that wood is a porous material, which allows an exchange of gases with the outside. This is dependent on the wood grain size, which may vary among species, age and forest of origin [57]. Other factors affecting the gas exchange capacity of the barrel are the pressure drop generated within the barrel, the formation of a headspace, wood anatomy, the different oxygen entry routes, the role of wood moisture content and soluble ET, and the effect of barrel toasting on cooperage. It can also exchange gases and other materials by means of diffusion, both processes happening simultaneously [5]. The first investigation that studied the amount of oxygen that enters a barrel full of wine in a year estimated an amount of OTR between 15 and 45 mg/L per year [58]. Subsequent studies discussed the mechanism of how transfer occurs considering that it is a dynamic process intervening various interrelated factors. Among all them, the impregnation of wood, the formation of reduced pressure inside the barrel, the type of seal, wood and toast performed in cooperage play determinant roles [5]. Moreover, wood also absorbs wine due to the impregnation of the barrel walls. This is not the only factor that reduces the volume of wine since part of it is evaporated through the pores. All this causes barrel deformation over time as well as the loss of properties, which makes barrels items of limited shelf life [5]. It should also be noted that barrels contain a head space that is filled with air before closing them. During the aging process the level of oxygen is decreased. Wine degassing is the main agent that influences the composition of the gas in the headspace [5]. Due to the degassing, a gradient of dissolved oxygen is generated, having lower levels on the surface and the greatest one at the bottom of the barrel [18]. Moreover, depending on the wood used in barrel production, differentiated characteristics are acquired by wine (texture, flavor, aroma). In addition, the wood of the inner side of the barrels can act as a means of inoculation as demonstrated with lambic beer. These microorganisms would help establishing a stable microbial community, which would result in a higher homogeneity of the fermentation profile between batches. As for the type and variety of microorganisms, it will depend on the internal surface of the barrel and, consequently, on the age, thickness and porosity of the wood [59].

Despite its many advantages, barrels have several drawbacks. Barrels are expensive to produce, have limited lifetime and with each batch, there is an increased probability of growth of spoilage yeasts as in the case of the well-known *Brettanomyces* sp. [25]. Sanitation procedures between batches contribute to shorten the barrel’s lifetime too [48,60]. The lifetime extension of a barrel varies depending on its intended purpose. If it is dedicated to the transfer (sediment separation) it can be used up to 40 years; but if instead its use is for the contribution of aromas and flavors, its lifetime is drastically shortened to 8 years. Nevertheless, a subsequent bottle aging phase is always necessary. As all these factors influence the final quality of the wine, alternatives to barrels are being developed to carry out the wine-aging process and obtain the most similar aged wines in a more affordable and efficient way [10].

## 3. Compounds Present in the Wood That Affect the Characteristics of the Wine

Wood barrel aging improves not only color and mouthfeel, but also increases aroma complexity due to the extraction of compounds present in the wood. These compounds include cellulose, hemicellulose, lignin, acids, sugars, terpenes, volatile phenols and lactones, being compounds of very different molecular weights. Aging in wooden barrels is a process used to stabilize the color and enrich the sensorial characteristics of wine. Therefore, an aging period in the wooden barrel is required to attain sensory fullness and high quality.

From a sensory point of view, the most important compounds are the following: compounds that provide coconut and woody aroma (oak lactones, this is, cis and trans forms of β-Methyl-γ-octalactones), smoky aroma (guaiacol and 4-Methylguaiacol), green wood (vanillin), almond (furfural compounds) or spicy aromas (eugenol). Among these compounds, oak lactones are one of the most important to the sensory characteristics of wine along with eugenol and vanillin. In fact, lactones has been identified in other aged alcoholic beverages such as brandy or whisky and wine, so they are commonly known as “whisky”, “Quercus” or “oak” lactones [61,62] Other important compounds released from the wood are ET, which protect wine from oxidation due to their potent antioxidant properties [25].

A study that observed the aging of the wine using different woods showed that the wood affects to 41 aromas of the wine, 11 of them depending solely on the type of wood used. This is due to a series of processes that take place during the oxidative phase: oxidation of wine alcohols and amino acids, microbiological formation of ethyl phenols, sorption processes and condensation of acetaldehyde with polyphenols. The contact time between the wine and the wood also plays a fundamental role and compounds more proximal to the contact surface will be released at a higher rate. Some compounds present in all woods are linear γ- and δ-Lactones, β-Damascenone and ionones [63]. Aging duration is highly variable depending on wine′s origin, type and quality. The age of the barrels is also important as aged wines for a short period (6–9 months) show quite a big difference in the concentration of most of the oak wood compounds between wine aged in new barrels and wine aged in once-used barrels. This difference decreases in long-term aging (12–15 months). The compounds which become more exhausted from barrel use are usually furanic aldehydes, phenolic alcohols, phenolic aldehydes and oak-lactones [64]. In another study that used acacia, chestnut, cherry, mulberry and oak wood, it was shown that mulberry showed significant decreases of fruity-note ethyl esters and ethyl-guaiacol and a great cession of ethyl-phenol (horsey-odor defect), whereas cherry encouraged the highest polyphenol oxidation, making it less suitable for long aging [65]. It is worth noting that ethyl-phenol (4-Ethylphenol) and other volatile phenols like 4-Ethylguaicol or 4-Ethylcatechol in wines are exclusively produced by the spoilage yeast *Brettanomyces* sp. by degrading several PC of interest present in the aged wines. This compound is used as a biomarker for identifying wine spoilage and barrel contamination [66]. The presence or sensory levels of these compounds may be prevented by adding sulfites to the wine (inhibitor of this yeast growth) or filtrating the aged wine in order to remove them. In general, the method of choice is to add sulfites as they are cheaper and inhibit the growth of other potential spoilage microorganisms [48,66]. Other study analyzed hydroalcoholic extracts obtained from those types of wood and shows that the hydroalcoholic extracts of oak, chestnut and mulberry had higher total polyphenols content, followed by cherry, acacia and oak, respectively. Moreover, chestnut extracts showed the highest percentage of oxidizable compounds, followed by acacia, oak, mulberry and cherry. In all cases, the principal volatiles are benzene compounds containing a guaiacol residue, and high contents of C6–C18 fatty acids [65,67].

**Figure 2:**
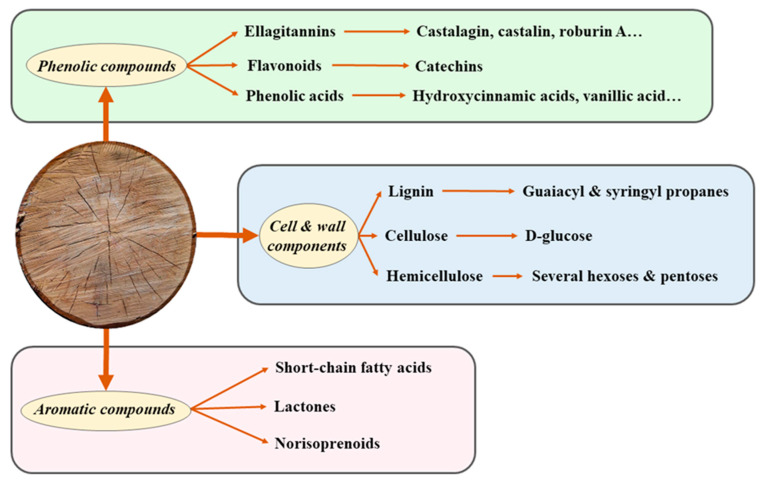
Major compounds of interest released from oak wood during wine aging. These compounds are common to the *Quercus* genus [54,67].

Therefore, alcoholic beverages quality will depend on the quality of the wood. Generally, to analyze this quality, research focuses on the study of PC and oak aroma volatile compounds. The profile of these compounds in aged wine provides an insight about the wood used, the aging time and whether it was aged in a barrel or using chips [68]. However, wood also provides other compounds of interest and less studied such as cellulose, hemicellulose and lignin. They are involved in several processes such as seasoning, burning or toasting. Seasoning and toasting generally exert a loss in ETand AC [69] A summary of the major compounds of interest released from oak wood during wine aging using *Quercus* genus as an example is shown in Figure 2. Hemicelluloses and lignin seem to be sources of compounds of interest by liberating free sugars or even some aromatic precursors, mainly during alcoholic beverage oak aging, whereas cellulose due to its crystalline structure, undergoes only few chemical degradations or modifications [70]. However, toasting may degrade cellulosic compounds and, in some cases, even increase the concentration of some AC (aldehyde and volatile PC or lactones) at low toasting intensity, but the available data on this issue is still somewhat contradictory [57,71] Furthermore, it is known that the organoleptic characteristics will not only depend on the type of wood but also where the wood comes from. This is because wines express a metabologeographic signature of the forest location where oaks of the barrel in which they were aged have grown due to compounds like lactones or nonvolatile ET [72].

The contribution of PC and tannins by wood has other advantages such as the increase of the antioxidant activity, being all their properties modulated by the kind of wood and the toasting level of the barrel. The aging process is accelerated in the case of *Castanea sativa, Q. pyrenaica* and *Q. robur*, with higher intensities of vanilla aroma and higher antioxidant activity. Other species of oak (*Q. petraea* and *Q. alba*) provide lower contents of PC and less intense related properties [73]. The effect of toasting method and forest origin was also studied in Merlot wine during 1-year barrel maturation. It was demonstrated that watering process during toasting enhances furanic compounds, vanillin and oak lactones extraction, whereas toasting barrel head pieces may lead to eugenol and ET degradation. Wine from lightly toasted barrels is perceived as less sweet, bitter and more astringent [74].

In cases where wine is aged with chips instead of in barrels, representative organoleptic characteristics of this process are also appreciated. In this case, long times (3 months) increase the concentration of cis-whiskey lactone and guaiacol in American oak-treated wine samples. For wines aged with French oak chips, higher concentrations of furfural, 5-Methylfurfural, 4-Vinylguaiacol and trans-whiskey lactone are achieved. The increased presence of chemical compounds in wine aged with French oak chips generated prominent smoky, licorice and toasty aromas, whereas in wines aged with American oak chips, notes of vanilla, toasty and cacao aromas were noticed. Moreover, red wines aged with American and French oak chips were discriminated by chemometric analysis, which confirmed the evolution of AC [75]. Furthermore, contact between wood chip extracts and grape skin isolated anthocyanin extracts induced a decrease of color intensity (particularly red color), and the anthocyanin content in the different experimental synthetic wine solutions studied [76].

## 4. Oxidative Aging Process

**Figure 3:**
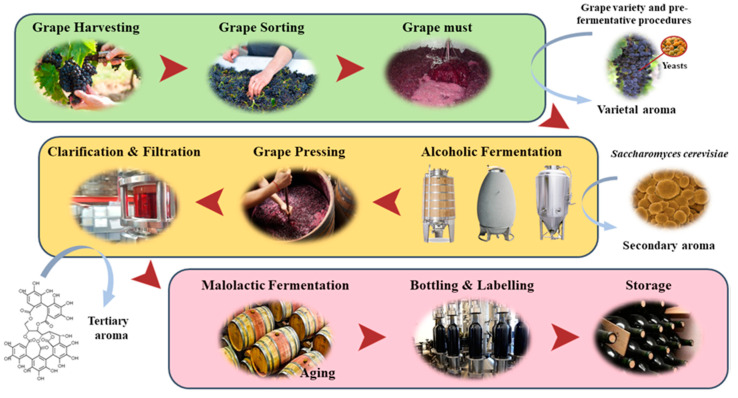
Production process of a red wine subjected to barrel aging. This process corresponds to the general winemaking process of red wines. In the case of white wines, the pressing step is carried out before alcoholic fermentation.

Given all the previous information and the history and economic importance of aging wines, the aging process can be considered as a fundamental stage in the winemaking process (Figure 3) of certain types of wines, characterized by specific organoleptic characteristics. The stability of a wine during storage is dependent on its chemical composition, which is continuously changing mainly due to factors such as temperature, light, bottle position, oxygen content and storage time [77]. Aging stage is the phase with greatest changes in chemical composition, mainly in PC composition and their abundance, as they are generally quite unstable and undergo oxidation, polymerization and co-pigmentation processes during aging with consequent changes in their antioxidant activity. As mentioned in previous sections, aging can be divided into two phases: wood phase (oxidative) that usually occurs in barrels and bottle phase (reductive) [10].

At this last phase, wine is bottled. This second phase takes place in airtight containers that prevent oxygen from entering. Therefore, the main changes are due to the degree of oxygen exposure in the bottle. In some cases, a prior clarification process is necessary, and bottling must be carried out in containers able to protect PC (especially hydroxycinnamic acids) and pigments from light [78]. At this stage, the stability of the bottled wine depends on different factors such as storage temperature (variable, constant and cold), bottle position (horizontal or vertical), time of storage (3, 6, 9, and 12 months) and variety [77]. Moreover, the changes produced during the reductive phase in bottle are dependent of the previous phase conditions. Therefore, as barrels are the main actors at the oxidative stage, the principal reactions that take place in this phase of the aging process and the different techniques apart from barrel aging are shown below.

### 4.1. Oxidative Aging in Barrel

As aforementioned, oxygen transfer is a fundamental requirement for aging. For this purpose, wine is settled into containers that must allow a certain oxygen transference. Historically, the most employed recipients for these purposes have been wood barrels. Therefore, the main exchanges that take place at this stage will be determined by the incorporation of oxygen through the wood (16% own wood, 63% joints, 21% elusive), by the surface (fillings) and racking and by the transfer of wood compounds and evaporation losses [79]. To correctly control this process, factors such as botanical and geographical origin of the wood, seasoning, the degree of toasting and the number of times the barrel was previously used must be taken into account [80]. For instance, the tree species is determinant for the OTR. Although it was believed that the oxygen that participated during the aging phase was contained in the air at the top of the barrel or was incorporated during barrel transfer and filling, it was found that the OTR also depends on the type of wood. This process is determined by the thickness of the wood layer, the permeability of the wood and the oxygen concentration difference between both sides, as well as the anatomical structure of the wood. In this way, a recent study found that American oak barrels allowed a higher oxygenation rate than French ones [81]. Oak barrels are widely used due to the numerous micropores that allow the continuous and moderate intake of oxygen, gradually integrated into the wine. The oxygen transference provided by the barrel will decrease over time and with each use, since the wood micropores will get progressively obstructed [5]. The stave gaps, the wood structure and the barrels’ bunghole are the main passage zones for oxygen [82]. The barrels can be made of various species, but oak barrels are the most used, due to the aroma attributes and the efficiency they provide. They are usually distinguished between French oak and American oak, and there are also different sizes available, being the most common 225 L (Bordeaux) and 300 L (Burgundy) barrels [5].

At this stage, aroma becomes more complex and/or intense due to the release of different compounds from the wood. Among the released compounds, some of them are responsible for texture and astringency changes, such as the ET. In this phase, the color is also stabilized due to the reactions occurring between anthocyanins and proanthocyanins [80]. The increase in color stability is mainly due to the entry of small amounts of air through the wood pores and the barrel lid, making possible a natural micro-oxygenation that improves polymerization and condensation reactions between flavonoid compounds and directly affect wine’s color and astringency, modifying their organoleptic characteristics [83]. However, the phenolic composition of wines (and so, its color) in oak barrels changes during oxidation processes and by incorporating derived phenolics extracted of wood, such as gallic acid, syringic acid, vanillic acid, ferulic acid, ellagic acid and ET, which provide desirable cofactors for copigmentation processes with anthocyanins [82]. This effect is changing continuously and will depend on the barrel used. For example, white wines have a tendency for a lesser increase of the browning potential index values in new oak wood barrels, being browning a serious problem for white wines, this is because of their high content in PC. As they can be easily oxidized to quinones, they can polymerize forming macromolecules with a yellow-brown hue [84]. Moreover, compounds derived from wood such as ET and ellagic acid are quicker absorbers of oxygen, facilitating the hydroperoxidation of wine constituents and thus acting as protective agents against color changes especially associated with the browning process. In addition, during this stage, a series of processes common to all wines (even if they are not aged in barrels) will also take place, such as sedimentation of the precipitated compounds present in wine, which results in a clarification of the wine and therefore, a lower degree of turbidity [80].

### 4.2. Oxidative Aging with Alternative Systems

Although the barrels release desirable compounds to the wine and allow a slight oxygenation (both desirable characteristics), their renovation is very expensive. To solve this problem, other artificial or alternative systems have been developed. The aging of wines with alternative systems can be carried out by three main different approaches: by the addition of wood pieces to wine, by combining these pieces with micro-oxygenation or by wine aging on lees [85]. The first system usually employs wood pieces of small size or “chips” that stay submerged on wine with the aim of giving aged wine properties that recall those of wine aged in barrels. The obtained results will vary depending of the piece size (which may be as large as a barrel stave), wood origin (usually, oak species) and the degree of toasting [86]. In the case of oak, pieces can be distinguished in tank staves, oak chips, portions of wood called cubes or oak beans, oak powder, pieces of granulated wood (pencil shavings or granulates), oak wood cut as dominoes (dominoes), and square pieces (blocks or segments) [87].

Regarding the production procedure, pieces are put in a chamber acting as an infusion, as they get soaked with the wine permitting the transference of different compounds like those of barrels to occur. Despite the attempt to imitate traditional techniques, this new system results in significantly different products than those obtained when wine is aged in oak barrels. After carrying out an analysis of volatile PC, it is possible to distinguish wines produced with oak chips from those of barrels due to the concentration of syringaldehyde, vanillin, guaiacol and furfural. In this study, other factors such as wood chips size were assessed, but generally, it was found that concentrations of these compounds when using oak chips were equal to or higher than when using barrel aging [88]. Other studies have been developed in this regard and found that greatest changes in composition were related to anthocyanin compounds, being higher using oak barrels than using oak chips [89]. Moreover, other factors such as toasting can influence the result when using this alternative system. For instance, wines from medium toast wood chips scored higher woody, vegetative and smoky aromas and flavors but also bitter taste and astringent mouthfeel. After 14 days they had the highest headspace concentrations of furfural and cis oak lactone. Wood-related notes were ranked from heavily and lightly toasted chips, barrel and steel control [90]. Although artificial systems do not allow obtaining the same organoleptic characteristics, different studies show that oak chips can be a good choice in the case of the elaboration of young wines with slight olfactory and gustative wood notes quite similar to wines aged in new barrels for short periods of time (about three months) [89]. Hence, this methodology has proven to be less expensive, save processing time and be more sustainable while it is capable of producing products of similar quality in comparison with barrel aging due to its enhanced transfer and incorporation of volatile compounds with woody notes [85]. For instance, a study researched the effect of artificial systems (staves and chips) and conventional barrels on aged wine and showed that wines previously treated with artificial systems underwent a quicker aging in bottle, with a quicker loss of anthocyanins and a higher number of polymerizations than the wine aged in barrels. Significant differences were also found among wines produced with different barrels, noting that French oak wood suffered a slightly lower loss of anthocyanins than those aged with Hungarian (*Quercus frainetto*) and American oak (*Quercus alba*) wood [91].

The second system is referred to the coupling of two techniques, the use of wood pieces together with micro-oxygenation that releases small amounts of oxygen, simulating the aging process in barrels in an artificial and controlled manner. This technique is usually applied to improve some processes as color stabilization and strengthen as well as to enhance the organoleptic properties of wine by the modification mainly of its tasty and aromatic characteristics. On this process, the most influential parameter is oxygen dosing, so dosing is applied trying to replicate the model of a wood barrel, even monitoring its concentration in some cases [85].

Finally, for white or sparkling wines, lees that remain after winemaking process of storage can be used to improve the process of aging. Lees generally consists of cell walls resulting from yeast autolysis and in minor proportion, of some precipitated inorganic compounds and tartaric acid. This aging option is coupled with either barrel aging or aging carried out in other containers, such as stainless tanks and large cooperage systems. The contact of the lees and the wine during the aging process can improve its sensory profile and characteristics as color stability, reduce bitterness and astringency or modify its organoleptic characteristics, among others. Therefore, it can improve the chances of obtaining an improved wine by alternative methods while maintaining reproducibility among batches of similar quality [85].

### 4.3. Accelerated Aging

This technology involves different techniques directed towards the acceleration of wine aging by means of application of micro-oxygenation or the application of some physical methods [85]. In many cases, it consists of applying optimized temperature and air conditions to shorten the time needed to produce a certain type of wine. In this way, some costs are reduced. As mentioned before, by subjecting a young wine to micro-oxygenation, a series of technological advantages are achieved, namely, color stabilization, strengthening of red color in red wine, enhanced health of yeasts during alcoholic fermentation taste and structure improvement of wine, modification of aromatic characters of wine and removal of undesired flavors and off-odors [38]. To achieve all these advantages, another option is to use wood fragments due to the compounds that wood releases when wine is not aged in wood barrels [92]. The parameters that most influence this process are free SO_2_, ethanol, color indictors, PC and dissolved oxygen available [93]. As aging involves high times and costs, several patents have been developed for the purpose of shortening these times. One of them consists in attaching a device to the aging barrel that induces movement of wine so that the liquid is more in contact with the surface of the container and with the air [94]. Other designs do not need any agitation mechanisms to be attached but only decrease the size of the pieces of wood until they are pulverized (<1 mm) in enough quantity to achieve equivalent aging in one-tenth to one-hundredth of the time required for traditional barrel aging [95]. Similar results can be achieved with the use of physical methods like low frequency polarized pulsating magnetic fields in the order of 50 kHz [96]. Other physical methods that enhance oxidation include ultrasonic waves, gamma rays, electric fields and nanogold photocatalysis [85]. An ultrasound bath system was patented in the USA, which managed to not only speed up the process but maintain the quality of the wine for long periods extending their shelf life [97]. The flowing system can also be used for aging wine made from some materials but not all (maize). In this process, wines are treated with ultrasounds when they pass through the atomizer orifice [98]. A system developed by the University of Cádiz is a practical example of this. It consists of an ultrasonic system with aeration where wine in contact with oak chips is introduced into an ultrasonic device connected to a larger chamber. At this chamber, oxygenation takes place due to a continuous flux of oxygen [99]. Gamma irradiation was also studied for aging wines not made from grapes (e.g., rice wine) with good results. However, further studies are needed as it might present toxicity problems [100]. Electric fields enhance the extraction of PC and accelerate aging process, showing that the optimal conditions for certain types of young red wines are 600 V/cm and treatment time 3 min [101]. Nanogold catalysts promote the formation of free hydroxyl radicals, which can accelerate chemical reactions and which have been used already in accelerating the maturation of young sorghum spirits [102]. As for high-pressure techniques, they are still in preliminary studies. Although applying this type of aging is intended to obtain wines similar to those obtained with non-accelerated aging, during the process, a series of volatile compounds (C6 alcohols, dioxolanes, β-Damascenone, γ-Butyrolactone and 1,2-Dihydri-1,1,6-Trimethylnaphtalene) are developed and allow them to be differentiated [103].

## 5. Comparison of Different Aging Technologies

In recent years, the feasibility of different materials and methodologies that could be employed to produce quality aged wines has been explored. Regarding all the different aging technologies mentioned above, they are compared and explained below.

The most efficient way of mimicking barrel aging in terms of oxygenation and aroma is a combination of micro-oxygenation and wood chips. The wood chips can be toasted and present different size, the smaller they are, the easier the wine soaks them and the easier ET are transferred, as the active surface is higher [104]. The species used to produce the wood chips may be solely oak (French or American) or a mixture of oak with other species such as chestnut or cherry to obtain more characteristic aromas. This methodology is most commonly used in stainless steel tanks as it is a non-porous material, but the use of chips may be paired with clay, concrete, stoneware or polyethylene vessels since these materials allow oxygen transference [44]. Aging wine in clay or concrete vessels is experiencing a negative trend in its use due to a renewed interest in favor of wood barrels. Given the porous structure of these materials, they allow an adequate oxygenation of wines, and the resulting acquired complexity that comes with wine aging [105]. However, as it would be expected, these materials do not present any PC or ET that can be transferred to aged wine. Nonetheless, they are much more affordable and feasible to sanitize without compromising any properties related to the aging process. Nevertheless, using some of these materials such as concrete or clay, especially this last one, confers the aged wine a mineral and earthy flavor, which some consumers find appealing as it adds a wider diversity of tastes [106]. The aging vessels can also be made of high-density polyethylene, a porous material that allows oxygenation while being highly affordable [107]. However, the oxygen transfer distribution is not equal in all the containment surfaces, as the sections near the gaseous phase of the volume show a higher OTR. Aging wine in this kind of vessels could be paired with the use of wood chips to add new flavors and a certain degree of micro-oxygenation, if the OTR is not among desired levels [18]. The use of these materials and methodologies gives off aged wines of high quality and similar aroma to traditional ones [44]. Most importantly, because of the lower investment required in comparison with traditionally aged wines, the retail price is more affordable, which makes them more accessible to consumers [17], although the resultant phenolic and aromatic profile of these wines is sensibly different. Wine experts can tell the difference in taste, mouthfeel and aroma, while the aging method used can also be determined with chemical analysis on PC, ET and AC profile, as these compounds tend to be lower in wines not aged in wood barrels [68,86].

Other practices focus on the bottle aging phase, trying to reach new and unique aromas. Bottle storage is usually carried out in underground cellars because it allows to preserve wine from light exposure, drastic temperature and moisture changes [108]. Different researchers have analyzed the effects of bottle aging and storage conditions on the properties of aged wine, whether focusing on the cap material, bottle position or stability of chemical compounds [109]. An innovative approach to enhance bottle aging has been made by storing sealed bottles underwater. The Spanish company “Crusoe Treasure” was one of the first cellars to use this new approach [110]. These wines are kept 20 m under sea level for at least 6 months after barrel aging and the resulting wine achieves a rather salty aroma and a significant retail price. This new methodology is also being explored by other cellars around the world, but for the moment, there is no publicly available data on the process or chemical composition of these wines aged underwater.

## 6. Species Used in Cooperage

As it has been described in previous sections, the contact between wine and the wood surface leads to significant changes in the wine, which entails a change in its quality, both in greater aromatic and flavor complexity, as well as greater stability. Altogether, these facts explain the high demand for wood barrels. However, after a few years of use, it is necessary to replace them because the barrel gradually loses PC, AC and ET that can be transferred to the wine, and the oxygenation rate lowers through batches [49]. Oak remains as the main species used for cooperage, but the increasing demand for this wood has prompted other species to be used for barrel manufacture. This tendency also involves a higher diversity of the resulting aged wines due to the different characteristics provided by each type of wood. A summary of the main species (traditional and non-traditional) used in wine cooperage is shown in Table 1 while species used for maturation of other alcoholic beverages is shown on Table 2.

### 6.1. Conventional

During the aging process in wooden barrels, wine undergoes significant modifications because of several phenomena. Mainly, through the release of wood extractable compounds into the wine, being the most important those of low molecular weight which exert influence on the color, aroma and flavor of the aged wine. In turn, this release of compounds will be determined by the wood botanical species and the cooperage process (manufacture, seasoning, dimensions) [123].

Since the 16th century, the most used species in cooperage are oak and chestnut [120]. As a result, oak wood in wine aging has been deeply studied. Among the diversity of oak species, French and American oaks are the most extensively employed. Even so, other European countries grow and use some indigenous oak species for barrel manufacture, like Spain, for example. Both are made from the same genus of white oak, but there is where their similarities end. Therefore, depending on the material, the resulting wine will have different characteristics. Both American oak and French oak contribute to aromas, flavors and tannin composition to a wine. As previously mentioned, barrels produced with French oak are up to 2-4 times more expensive than American ones. This is due to the more irregular and porous grain structure that leads to greater losses in the gluing of French oak. American oak is denser, so it can be sawn instead of hand-split which involves less labor and expense. [52]. The different structure of the wood between these oaks leads to wine producers to use these woods for diverse types of aged wines.

American oak or white oak (*Q. alba*) is original of the East Coast of the United States of America. *Q. petraea* or *Q. robur* are the species classified as French Oak. Among these two lasts, *Q. petraea* is considered better for winemaking. As oaks have been grown and used for many centuries for wine aging, France has some of the most important oak forests such as Allier, Nevers and Tronçais (all in central France), Vosges in the northeast and Limousin, which is more westerly, near the Cognac region. Of these five, Limousin is the only forest where *Q. robur* grows. Depending on the region, they will provide different characteristics. This way, wines matured in Vosges oak barrels are the ones with higher levels of cis and trans-oak lactone and eugenol and limousine oak barrels the lower ones. However, the Limousin-oaked wines were still richer in these compounds than the American-oaked wines [127]. Another type of compounds that allows them to be differentiated are phenols. Different studies showed that French oak barrels provide 2.5 times more phenols than American ones, being in both types 87.7% of the extracted non-flavonoid phenols [128]. This observation agrees with the results of other researchers. It is also observed that in wines stored in American oak barrels, the evaporation of ethanol is greater, being even higher during the first month of aging. Anthocyanins content is also decreasing, resulting in a higher color intensity at the beginning of the process due to its polymerization [129]. In addition, some authors have suggested that the note “vanilla” used by the tasters is probably not totally due to the presence of vanillin and would be related to cis-whiskylactone content of wines. American oak is sweeter and contains more vanillin compounds, providing an overall sweeter flavor to the wine [130]. Compositional differences due to variances in soil, climate and growth habit account for regional differences in France. Moreover, the level of PC in the wood tends to increase with tree’s age and size. Another factor that will also increase the differences among barrels is the different methods of producing them (air-drying, kiln-drying and the level of toasting or charring on the inside of the barrel) [128].

A different study compared the evolution of bottled wine after being subjected to a maturation process in barrels made with different woods and showed that wines aged in Spanish and French oak wood barrels, after 24 months stored in bottle, have similar characteristics. However, they are significantly different in comparison to those aged in barrels made of American oak. Results are more homogeneous the longer the wines are left in the bottles [131].

### 6.2. Non-Conventional

Due to the need for finding other suitable sources of wood to carry out barrel aging, the chemical profile and suitability of other less used oak species has been studied. These species include *Q. pyrenaica, Q. faginea, Q. frainetto, Q. oocarpa, Q. humboldtii, Q. serrata, Q. mongolica* or *Q. denta*. Particularly, *Q. pyrenaica* is used mostly in the form of chips or staves, resulting in wines of optimal final characteristics [132]. Different comparative studies guarantee that the resulting wines have characteristics similar to those wines made in French oak barrels, having a lesser degree of similarity with those produced in American oak barrels [133]. Furthermore, a study showed that at sensory level, consumers prefer wines produced with *Q. pyrenaica* rather than French or American oak woods [117] *Q. faginea* is a good alternative species for cooperage due to its antioxidant properties [119]. *Q. humboldtii* sensorial analysis presents no negative comments from the tasters about the macerated wine [121]. Moreover, few significant differences in the sensorial analysis were observed in these wines compared to those aged with traditional oaks. Therefore, *Q. humboldtii* oak has an interesting oenological potential as an alternative species for coopering [122].

The use of other species that are not of the oak family is also possible and will also give a special character to the resulting wines. Among these, some can be highlighted: *Castanea sativa, Robinia pseudoacacia, Prunus avium, P. cereasus, Fraxinus excelsior, F. americana, Morus alba* and *M. nigra*, allowing a wider spectrum of wood fitted for wine aging in the future as further research is being carried out [2]. However, it is more common to combine the unique aromas of these woods with oak by aging with wood chips [126].

### 6.3. Legislation

Despite the great variety of woods that could be used, only a few are allowed because they are the only ones that do not contribute unwanted compounds to the final product. These are those of the species oak and chestnut, which are the only ones approved by the International Organization of Vine and Wine (OIV) (Resolution OENO 4/2005). As the maturation process in barrels involves long periods and high costs, alternative techniques were developed to counteract these disadvantages. This is the case of the addition of wood chips, a technique that has been used for more than 15 years worldwide and whose use has even been regulated by the European Union. This maturation practice is regulated by the International Oenological Codex of the International Organization of Vine and Wine (OIV) (OENO 9/2001) and by the Official Journal of the European Union (CE 1507/2006). This regulation states that the use of wood chips is limited to the exclusive use of Quercus species. Moreover, in terms of the specific regulations, each country counts with different specifications and definitions. For instance, in the case of Spanish legislation, article 18 of Real Decreto 1363/2011, of 7th October, by which the Community regulations on labeling, presentation and identification of details of wine products is developed, the term “roble” (oak in Spanish) refers only to those wines aged in oak barrels while the term “barrica” (barrel in Spanish) can be used when the container is of any kind of wood [134]. Thus, it is an important specification since many studies are focusing on other types of woods for wine aging that, despite not being currently used, have shown high potential of application [7].

In addition, for the selling of wine barrels, there are also some requirements such as indicating the time of aging in wood and that barrels cannot be containers of more than 600 L. Notwithstanding this, the indication “Barrel fermented” may be used for wines whose fermentation has taken place in barrels, without it being necessary to indicate the length of time in this case [134].

According to the OIV [6], the use of wood shavings in the aging process of wine is allowed in order to introduce the characteristics of certain oak wood constituents into wine. However, these chips have to fulfil certain characteristics as the pieces of oak wood shall comply with the prescriptions of the International Oenological Codex, and they cannot be charred, including the surface.

## 7. Conclusions

Aged wines constitute a great proportion of the global wine market, holding both a high traditional and economic value. The aging process is an essential step in the winemaking process as it exerts notable changes in the chemical and aromatic profile of wines, and therefore, it is responsible for the production of high-quality wines. In this context, this article focused on the importance of the oxidative aging stage, particularly the role of barrels. These can be considered as an active vessel in the winemaking process. Hence, compounds transferred to wine depend on the species used for making barrels, which have traditionally been French and American oak. However, new opportunities are being investigated in order to develop the use of other types of woods. Nevertheless, barrels show a series of drawbacks such as shelf life, length of time and high production costs, so the challenge now is to develop alternative techniques for reducing production costs, optimizing sanitation procedures and accelerating the process. On the other hand, other approaches are currently widely employed such as the use of alternative materials or the addition of wood chips combined with micro-oxygenation as well as the application of other physical techniques to accelerate this process. Furthermore, research still continues on understanding the kinetics of oxygen in aging and the identification and characterization of AC.

Therefore, new approaches go in two directions: the search of new woods and new related organoleptic attributes and the transformation of barrel aging into a more affordable and feasible process, especially the development of alternative systems able to imitate or improve the sensory profile at the same time that process is accelerated and costs are reduced. For this purpose, research is now focusing on barrel simulation to completely understand and monitor the process and thus be able of replicate it. However, barrels still are the most recognized technique, and they are not likely to be completely abandoned in the coming years.

## Figures and Tables

**Table 1:** Main species used in cooperage. Characteristics of the wood and properties of the resulting wine.

Species and Distribution	Characteristics	Chemical Composition	Results	Ref.
**Traditional Woods Used in Cooperage**				
American oak (*Quercus alba*) East USA	Regular sawing, few manufacturing losses, lower price (−0/−60%), very dense	Contribution to whiskey-lactones	Little risk of green taste, low tannin content, sugary character, fast wood intake	[111]
French oak (*Q. petraea* or *Q. robur)* North France	Branch removal necessary, large manufacturing losses, high price, medium density	Higher content in phenols and flavonoids	Green taste with too short drying, high tannin content, limited aromatic contribution, slow wood intake	[111]
**Non-traditional Woods in Cooperage from Oak Species**				
*Quercus pyrenaica* Western Atlantic–Mediterranean regions	Appropriate structural properties (mesh, grain, density, and permeability)	ET, low weight compounds and AC	Higher aromatic intensity and complexity. Woody, balsamic and cocoa notes. High levels of eugenol, guaiacol, cis-β-Methyl-γ-octalactone and other volatile phenols	[112,113,114,115]
*Quercus faginea* Iberian Peninsula and North Africa	White yellowish sapwood and brown yellowish heartwood. High density and considerable mechanical strength	Castalagin and vescalagin are the main ET	Wines related to trans-resveratrol, p-Hydroxybenzaldehyde, syringic acid, ellagic acid and 5–HMF	[116,117]
*Quercus frainetto* Balkan Peninsula, South Italy and Northwest Turkey	High durability, ultra-structure comparable to French oaks, lindens similar to *Q. alba*. Longer heating during taming due to their high density	High content in ET	High bitterness and particular and indefinable aromas. Both attributes can be cushioned by the natural drying and toasting of the wood	[118]
*Quercus oocarpa* South America	Ultra-structure comparable to French oaks with a clear succession of early and late wood, forming an annual growth	Monomers of ET	Regarding the gustatory aspect, it is similar to *Q. petraea*	[118]
*Quercus humboldtii* Colombia	Hard, heavy and easy to work	Most abundant phenolic acids, aldehydes and ET being the same as in *Q. alba* and *Q. petraea*. Phenolic composition closer to American ones	Balanced syringaldehyde/vanillin relationship. Higher concentrations of 5-Methylfurfural, guaiacol, isoeugenol, trans-Isoeugenol and syringol. Lower furfural, 5–HMF, trans-β-Methyl-γ-octalactone, and cis-β-Methyl-γ-octalactone content	[96,119,120]
**Untraditional Woods in Cooperage Different from Oak Species**				
*Castanea sativa* Southern Europe and Asia	The only species alongside *Quercus* that has been accepted for its use by the International Organisation of Vine and Wine (OIV)	Low content of oxidizable polyphenols (less suitable for prolonged aging)	Higher content of total PC and of low molecular weight compounds. Higher antioxidant activities. Vanilla notes	[65,121]
*Robinia pseudoacacia* USA, Europe	Cheap, hard and low porosity	Rich in mono and di-methoxyphenols, acetosyringone and ethyl vanillate. High content in simple volatile PC	Red wines with higher smoky, spicy and fruity notes	[7,122]
*Prunus (P. avium and P. cerasus)* Europe and western Asia	High porosity and oxygen permeation. Used for short aging times	Aromadendrin, naringenin, taxifolin, isosakuranetin, eriodictyol and prunin	Greater oxygen penetration through their staves	[65,68,123]
*Fraxinus* spp. Europe, Asia Minor, and North Africa	Moderately heavy, strong, rigid, hard and resistant to shocks	High content of 3-Ethyl and 3,5-Dimethylcyclotene, o-cresol, α-Methylcrotonalactone and vanillin. Low content of furanic derivatives	Less vanilla notes than oak	[7]
*Morus* spp. Asia, Africa, Europe, and North, Central, and South America	Tender, elastic, medium porosity, low release of compounds	Decrease in fruity-note ethyl esters and ethyl-guaiacol and the high cession of ethyl-phenol (a horsey-odor defect)	Hardly suitable for wine aging	[65,124]

**Table 2:** Species used for the maturation of other alcoholic beverages.

Specie	Origin	Main Uses	Characteristics	Ref.
*Q. alba*	America	Wine, bourbon, whisky, sherry.	High concentration of volatiles, low concentration of ET.	[125]
*Q. robur, Q. petraea*	France	Wine, brandy	Depends on the region	[125]
*Q. robur, Q.petracea, Q. petraea*	Eastern Europe	Wine, brandy, beer	High concentration of ET	[125]
*Q. dendata, Q. crispula, Q. mongolica*	Asia	Wine, brandy	Sweet taste	[125]
*Castanea sativa*	North Spain, Portugal	Cider, spirits	Valorization of a by-product of the lumber industry as wood chips	[126]
